# Meta-analysis of the efficacy and safety of vamorolone in Duchenne muscular dystrophy

**DOI:** 10.1007/s10072-024-07939-1

**Published:** 2024-12-24

**Authors:** Mohamed Said Ibrahim, Omar Ahmed Abdelwahab, Bashaer Elawfi, Fatmaelzahraa Yasser Ali, Sarah Amro, Shrouk F. Mohammed, Nour Shaheen, Ahmed Negida, Martin Arndt, Mido Max Hijazi, Jochen Schaefer, Timo Siepmann

**Affiliations:** 1 Dresden International University, Division of Medicine, Dresden, Germany; 2 Medical Research Group of Egypt, Cairo, Egypt; 3 Medical Research Group of Egypt, Negida Academy, Arlington, MA USA; 4 Faculty of Medicine, Al-Azhar University, Cairo, Egypt; 5 Faculty of Medicine, Sana’a University, Sana’a, Yemen; 6 Faculty of Medicine, Zagazig University, Zagazig, Egypt; 7 Al-Najah National University, Nablus, Palestine; 8 Faculty of Medicine, Alexandria University, Alexandria, Egypt; 9 School of Pharmacy and Biomedical Sciences, University of Portsmouth, Portsmount, UK; 10 Department of Global Health and Social Medicine, Harvard Medical School, Boston, MA USA; 11 Department of Neurology, Medical Faculty and University Hospital Carl Gustav Carus, TUD Dresden University of Technology, Dresden, Germany; 12 Department of Neurosurgery, Medical Faculty and University Hospital Carl Gustav Carus, TUD Dresden University of Technology, Dresden, Germany

**Keywords:** Vamorolone, Muscle, Steroid, Drug, 6MWT, TTRW, TTSTAND, TTCLIMB, NSAA

## Abstract

**Background:**

Duchenne muscular dystrophy (DMD) is a severe neuromuscular disorder, often leading to wheelchair dependence by age 13 with limited treatment options, largely relying on glucocorticosteroids. We assessed the efficacy and safety of vamorolone, a modified synthetic corticosteroid, for DMD.

**Methods:**

We performed a systematic review and meta-analysis using seven databases including prospective studies comparing vamorolone with glucocorticosteroids or placebo in DMD patients. We extracted data on efficacy and safety outcomes. We built fixed effects models to assess mean differences. (PROSPERO: CRD42023396908).

**Results:**

Out of 210 identified records, two study reports were included in meta-analysis providing data from 210 patients. Vamorolone at 2.0 mg/kg/day was associated with improvement time to climb four stairs velocity (MD = 0.05 95% CI [0.03 to 0.08] *P* = 0.0002), and time stand from supine velocity (MD = 0.07 95% CI [0.01 to 0.07] *P* = 0.007). A higher dose of 6.0 mg/kg/day was additionally associated with higher time to run/walk 10 m velocity (MD = 0.10 95% CI [-0.0.1 to 0.21] *P* = 0.07, I2 = 0%). Among these beneficial effects only improvement in time to climb four stairs velocity was sustained after a follow-up period of 30 months. Vamorolone did not inhibit growth but increased the risk of weight gain, suppression of adrenal function, and insulin resistance.

**Conclusion:**

The results of our systematic review and meta-analyis are suggestive of improved efficacy and safety of vamorolone for DMD compared to standard glucocorticosteroids but the external validity of these findings as well as the medication’s long-term effects remain to be determined.

**Supplementary Information:**

The online version contains supplementary material available at 10.1007/s10072-024-07939-1.

## Introduction

Duchenne muscular dystrophy (DMD) is a genetic neuromuscular disorder inherited in an x-linked recessive manner and caused by mutations in the dystrophin gene. This cytoskeletal protein is important for myofiber stability [1]. The prevalence of DMD is estimated to be 7.1 cases per 100,000 males worldwide and 2.8 cases per 100,000 in the general population [2]. DMD patients are diagnosed in early childhood and present with muscle weakness and delayed motor development. As the disease progresses, patients lose the ability to walk, and cardiac and respiratory function deteriorate [3]. Although gene replacement therapy, gene-editing strategies, cell-based treatments, and small-molecule drugs are being investigated, there is no definitive cure. Multidisciplinary care helps to slow disease progression and prolong life [4, 5].

Most DMD patients are treated with glucocorticosteroids which improve muscle function and delay loss of ambulation. However, there are many adverse events, such as increased bone fragility, frequent vertebral fractures, growth retardation, delayed puberty, metabolic disturbances, weight gain, adrenal suppression, mood and behavioural changes [4, 5].

Vamorolone is a steroidal anti-inflammatory drug which lacks the 11-hydroxy-carbonyl group but has the same affinity for glucocorticoid receptors as standard glucocorticosteroids [6]. However, the vamorolone-glucocorticosteroid receptor complex has lower gene transcriptional activity (“transactivation”) than traditional steroids [6, 7]. Unlike conventional steroids, vamorolone is a mineralocorticoid antagonist which aids in blood pressure regulation [7]. In contrast, multiple studies have shown that vamorolone improves membrane stabilization and retains anti-inflammatory NFκB-related inhibitory activities (“transrepression”). This benefits dystrophin stability while avoiding the side effects of glucocorticosteroids [8, 9].

The objective of this systematic review and meta-analysis is to evaluate the safety and efficacy of vamorolone in the treatment of patients with DMD.

## Materials and methods

We performed a systematic review and meta-analysis in compliance with the Preferred Reporting Items for Systematic Reviews and Meta-Analyses (PRISMA) statement and the Cochrane Handbook of Systematic Reviews and Meta-analysis of Interventions [10, 11]. The protocol of this review was registered on PROSPERO (CRD42023396908) prior to conduction.

### Eligibility criteria

Studies were included in our review if they satisfied the following criteria.

- **Population:** We included studies on patients with Duchenne Muscular Dystrophy.
- **Intervention:** We included studies in which the experimental (or exposed) group received vamorolone.
- **Comparator:** We included studies where the control group received glucocorticosteroids or placebo or no comparison.
- **Outcome:** We included studies reporting at least one of the following outcomes: *Efficacy outcomes:* (1) Six minutes walking test (6MWT) velocity (meters/second); (2) Time to run/walk (TTRW) 10 m velocity (meters/second); (3) Time to climb four stairs (TTCLIMB) velocity (event/second); (4) Time stand from supine (TTSTAND) velocity (event/second); (5) North star ambulatory assessment (NSAA) score (of 34). *Safety outcomes:* (1) Growth measures (mean height percentile for age and mean BMI z-score); (2) Biomarkers of bone turnover (Osteocalcin (ng/mL), procollagen type I propeptides (P1NP) (ng/mL), and type 1 collagen cross-linked C-telopeptide (CTX1) (pg/mL)); (3) Adrenal axis suppression (Morning cortisol and ACTH levels (μg/dL)); (4) Biomarker of muscle membrane damage (serum creatine kinase (CK) (U/L)); (5) Biomarkers for insulin resistance (fasting serum insulin and serum glucose); (6) Treatment emergent adverse events (TEAE).
- **Study design:** We included controlled or uncontrolled trials with patients allocated to receive vamorolone, no vamorolone, placebo, or other steroids in a random or non-random allocation manner. We considered blinded studies and open-label studies. We excluded studies whose data were not reliable for extraction and analysis, studies which were reported as abstracts only or theses, studies with incomplete text, and studies which were not published in the English language. We also excluded reviews, case reports, case series, and observational studies.

### Information sources and search strategy

We performed a comprehensive search of seven electronic databases (MEDLINE via PubMed, Scopus, Web of Science, Science Direct, EBSCO, Clinical trial gov, and Cochrane Library) until 10 November 2022 using the following MeSH terms and Boolean operators: (“Vamorolone” OR “VBP15”) AND (“Duchenne Muscular Dystrophy”). The full search string is provided in Supplementary Text 1. In addition, the references from these publications were checked for any additional information which could be eligible for this review. Literature search was performed by two independent reviewers (M.S.I. and O.A.A). Any disagreement was solved by consensus.

### Selection process

We used the Endnote software package (Clarivate Analytics, PA, USA) to delete duplicates. The collected references were screened in two steps: First, evaluation of the titles and abstracts to determine their relevance for this meta-analysis, and second, evaluation of the full-text versions of the identified articles. The selection process was conducted using the Rayyan platform by two independent investigators (M.S.I. and O.A.A) and any disagreement was solved by consensus [12].

### Data collection process and data items

Data were extracted to a uniform data extraction sheet. The extracted data included (1) Characteristics of the included studies in term of study ID, title, study design, country, population, interventions, doses, number of participants, treatment duration, and main findings; (2) Characteristics of the population of included studies in term of age, sex, weight, height, BMI, and baseline of 6MWT, TTRW velocity, TTCLIMB velocity, TTSTAND velocity, NSAA score, mean height percentile for age, and Mean BMI z-score; (3) Risk of bias domains; (4) Outcome measures as previously described. Data extraction was conducted by five authors independently (B.E., F.Y.A., S.A., S.F.M., and M.S.I.).

### Assessment of risk of bias

We used the Cochrane assessment tool 2 (ROB2) for randomized controlled trials to assess risk of bias [13]. The risk of bias assessment included the following domains: bias arising from the randomization process, bias due to deviations from intended interventions, bias due to missing outcome data, bias in the measurement of the outcome, bias in the selection of the reported result. The authors' judgments were categorized as "low risk," "high risk," or "some concerns" of bias. We used ROBINS-I tool for the non- randomized studies of interventions [14]. The risk of bias assessment in this tool included the following domains: bias due to confounding evidence, bias in the selection of study participants, bias in classification of interventions, bias due to deviations from intended interventions, bias due to missing data, bias in the measurement of outcomes, and bias in the selection of the reported results. We used the National Institutes of Health (NIH) tool for uncontrolled studies before and after clinical trials [15].

### Effect measures

In the present meta-analysis, we considered the following efficacy outcome measures:

- 6MWT: estimates the distance that a person can walk on a firm, flat surface in six minutes by meters [16].
- TTRW velocity: the total amount of time needed to walk 10 m measured by meters/second [17].
- TTCLIMB velocity: the total amount of time needed to climb 4 stairs measured by event/second [17].
- TTSTAND velocity: the total amount of time needed to stand up from a supine position measured by event/second [17].
- NSAA score: a 17-item scale used to assess the performance of a variety of functional skills on a scale from 0 (unable) to 1 (completes independently but with adjustments), and 2 (completed without compensation) [18].

An increase in any of these five outcome measures relates to clinical improvement.

Moreover, we considered the following safety outcome measures:

- Growth measures (mean height percentile for age and mean BMI)
- Biomarkers of bone turnover (Osteocalcin, P1NP, and CTX1). Osteocalcin and P1NP are measures of bone formation (decrease with glucocorticoid treatment), and CTX1 is a measure of bone resorption (increases with glucocorticoid treatment).
- Adrenal axis suppression (Morning cortisol and ACTH levels). Glucocorticoid treatment leads to decrease in both morning cortisol and ACTH levels.
- Biomarker of muscle membrane damage (serum CK). Serum CK elevation indicates muscle membrane damage caused by DMD.
- Biomarkers for insulin resistance (fasting serum insulin and serum glucose). Glucocorticoid treatment is associated with insulin resistance, which is determined by elevated fasting serum insulin and serum glucose.
- TEAE (treatment emergent adverse events).

### Synthesis methods

Since all included variables were continuous, the mean difference (MD) with 95% confidence intervals (CI) were calculated to estimate the difference in outcome measures between intervention and control groups using fixed effects model using the ReviewManager version 5.4.1 software package (The Cochrane Collaboration). Forest plots were generated.

### Assessment of heterogeneity

Statistical heterogeneity between studies was calculated using the Chi-square test (Cochrane Q test). Then, the chi-square statistic, Cochrane Q, was used to calculate the I-squared according to the equation: ${I}^{2}=\left(\left(Q-df\right)/Q\right)x100\%$. A chi-square *P* value less than 0.1 was considered as significant heterogeneity. I-square values ≥ 50% were indicative of high heterogeneity.

### Assessment of publication bias

We did not assess publication bias by Egger’s test via funnel plot asymmetry because publication bias assessment is unreliable for < 10 pooled studies [19].

## Results

### Literature search results

**Fig. 1:**
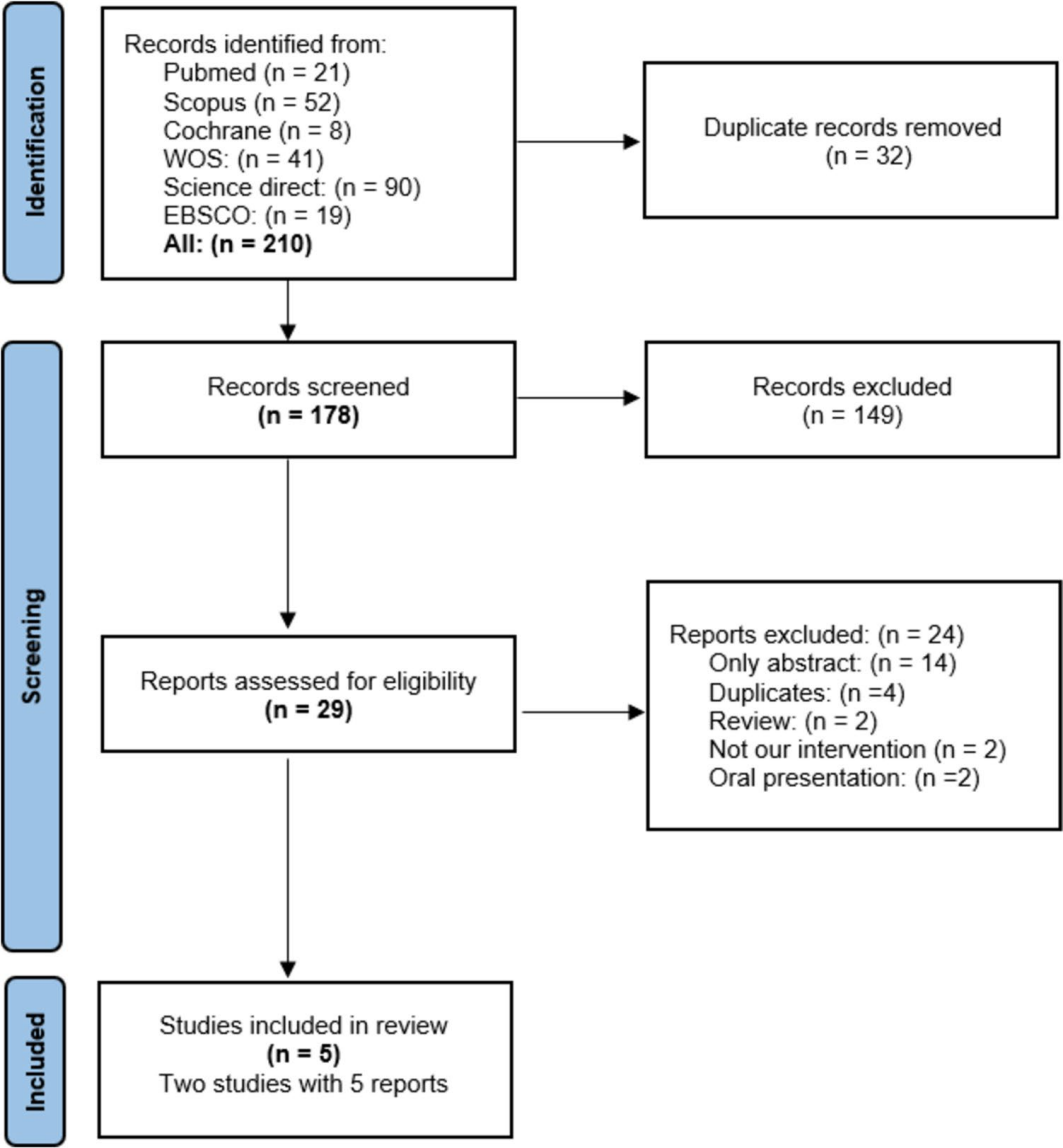
PRISMA flow diagram of studies' screening and selection. Two of the five studies included in the review were also included in meta-analysis after exclusion of those with overlapping study populations

Our literature search yielded 210 results. After reviewing the titles and abstracts, 29 articles were considered eligible. Only five publications were included in the systematic review. No further articles were included after manually searching the references of the listed studies. Figure 1 shows the PRISMA flow diagram of the study selection process.

### Characteristics and quality of the included studies

Five articles [8, 9, 20–22] were included in this systematic review. We excluded studies with identical study populations from meta-analysis. representing two studies with a total of 494 DMD patients (106 in the vamorolone groups and 388 in the control groups) [8, 9, 20–22]. A summary and baseline characteristics of the included studies are provided in Tables 1 and 2. Regarding the risk of bias, the study by Guglieri et al. 2022 [22] had a low risk of bias, the studies by Hoffman et al. 2019 [21], Smith et al. 2020 [8], and Mah et al. 2022 [20] each displayed a moderate risk of bias, and the study conducted by Conklin et al. 2018 [9] had a good quality as further detailed in Supplementary Table S1.

**Table 1:** Summary of the five included studies

Study ID	Design	Country	Intervention	Dose	No. of participants	Duration
Conklin 2018 [9]	Single arm clinical trial	US, Canada, Australia, Israel, Sweden, UK	Vamorolone	0.25 mg/kg/d	12	4 weeks
				0.75 mg/kg/d	12	
				2.0 mg/kg/d	12	
				6.0 mg/kg/d	12	
Hoffman 2019 [21]	Non-randomized controlled trial	US, Canada, Australia, Israel, Sweden, UK	Vamorolone	0.25 mg/kg/d	12	24 weeks
				0.75 mg/kg/d	12	
				2.0 mg/kg/d	12	
				6.0 mg/kg/d	12	
			CINRG DNHS corticosteroid-naïve	NA	31	
			CINRG prednisone treated	0.75 mg/kg/d	14	
Smith 2020 [8]	Non-randomized controlled trial	US, Canada, Australia, Israel, Sweden, UK	Vamolorone (VBP15-LTE group C + D	group C: 2.0 mg/kg/d; group D: 6.0 mg/kg/d	23	72 weeks
			CINRG DNHS corticosteroid-naïve	NA	19	
			CINRG DNHS corticosteroid- treated	NA	68	
			Prednisone (CINRG prednisone trial)	0.75 mg/kg/d	12	
Mah 2022 [20]	Non-randomized controlled trial	US, Canada, Australia, Israel, Sweden, UK	Higher-dose Vamorolone	2.0 mg/kg/d or greater dose (ie, 2 mg/kg/d and 6 mg/kg/d)	23	30 months
			GC in CINRG DNHS		75	
			GC in NorthStar UK Network		110	
Guglieri 2022 [22]	Randomized controlled trial	UK, US, Canada, Belgium, Czech Republic, Australia, Sweden	Vamorolone group 1	6 mg/kg per day	28	24 weeks
			Vamorolone group 2	2 mg/kg per day	30	
			Prednisone	0.75 mg/kg per day	31	
			placebo	NA	28	

*US,* United States of America; *UK,* United Kingdom; *CINRG DNHS,* Cooperative International Neuromuscular Research Group Duchenne Natural History; *CINRG,* Cooperative International Neuromuscular Research Group; *GC,* Glucocorticosteroid; *NA,* Not applicable

**Table 2:** Baseline characteristics of the included studies

Study ID	Age (years)	Height (cm)	Weight (kg)	BMI (kg/m^2^)	6MWT (m)	TTRW velocity (m/s)	TTCLIMB velocity (m/s)	TTSTAND velocity (events/s)	NSAA score	Height percentile for age	BMI z-score
Conklin 2018 [9]	5.2 (1.0)	NR	NR	NR	NR	NR	NR	6.1 (2.1)	NR	NR	NR
	4.8 (0.8)							5.1 (2.3)			
	4.7 (0.9)							5.3 (2.0)			
	4.8 (0.8)							5.9 (2.8)			
Hoffman 2019 [21]	5.2 (1.0)	NR	NR	17.4 (1.1)	316 (59)	1.6 (0.312)	0.2 (0.054)	0.18 (0.065)	19.0 (5.1)	NR	1.165 (0.6219)
	4.8 (0.8)			16.5 (1.5)	331 (53)	1.77 (0.367)	0.29 (0.147)	0.24 (0.09)	20.5 (5.6)		0.703 (1.0738)
	4.7 (0.9)			17.2 (0.8)	354 (65)	1.84 (0.347)	0.29 (0.164)	0.22 (0.082)	20.0 (4.9)		1.2 (0.5325)
	4.8 (0.8)			16.5 (1.0)	337 (63)	1.64 (0.279)	0.24 (0.086)	0.19 (0.056)	19.7 (4.9)		0.695 (0.7189)
	4.9 (0.8)			15.2 (1.6)	NR	1.64 (0.441)	0.21 (0.094)	0.20 (0.065)	NR		NR
	5.7 (0.7)			16.3 (1.7)	NR	NR	NR	NR	NR		0.44 (1.1607)
Smith 2020 [8]	5.2 (0.9)	107 (6.8)	19.5 (2.5)	17 (0.9)	343.2 (64.3)	1.735 (0.331)	0.266 (0.134)	0.206 (0.07)	19.9 (4.9)	29.19 (24.66)	1.03 (0.56)
	5.03 (0.55)	105.4 (5.1)	18.3 (2)	16.4 (0.9)	NR	1.619 (0.483)	0.218 (0.098)	0.202 (0.055)	NR	25.76 (21.37)	0.70 (0.58)
	5.96 (0.64)	109.2 (5.7)	20.6 (3.4)	17.2 (1.9)	NR	NR	NR	NR	25.76 (21.37)	20.09 (22.58)	0.98 (0.85)
	5.7 (0.66)	110.3 (6.8)	20.1 (3.5)	16.5 (1.9)	NR	NR	NR	NR	20.09 (22.58)	29.89 (29.15)	0.61 (1.27)
Mah 2022 [20]	5.83 (0.88)	111.80 (6.94)	21.98 (3.78)	17.68 (1.23)	377.9 (64.77)	1.90 (0.34)	0.31 (0.13)	0.25 (0.10)	29.89 (29.15)	32.26 (26.87)	1.28 (0.51)
	6.08 (0.81)	109.86 (6.86)	20.35 (3.55)	16.68 (1.55)	NR	1.91 (0.52)	0.32 (0.14)	0.25 (0.10)	32.26 (26.87)	19.88 (21.70)	0.65 (1.03)
	6.00 (0.77)	NR	NR	NR	NR	NR	NR	NR	26.63 (5.65)	NR	NR
Guglieri 2022 [22]	5.4 (0.9)	107 (7)	19 (3)	16.6 (1.4)	313 (56)	1.6 (0.4)	0.21 (0.09)	0.19 (0.06)	18.9 (4.1)	23 (25)	NR
	5.3 (0.9)	108 (9)	19 (4)	16.2 (1.2)	316 (58)	1.6 (0.3)	0.20 (0.05)	0.18 (0.05)	17.2 (4.7)	30 (29)	
	5.5 (0.9)	111 (6)	21 (3)	16.8 (1.3)	343 (56)	1.9 (0.4)	0.29 (0.11)	0.22 (0.06)	21.2 (5.5)	37 (29)	
	5.4 (0.8)	109 (9)	20 (3)	16.3 (1.1)	355 (78)	1.7 (0.3)	0.25 (0.09)	0.20 (0.06)	18.9 (5.3)	33 (29)	

Data are presented as means and standard deviations. *BMI,* body mass index; *TTRW,* time to run/walk; *TTCLIMB,* time to climb four stairs; *TTSTAND,* time stand from supine; *NSAA,* North Star Ambulatory Assessment; *NR,* not reported

### Efficacy

**Fig. 2:**
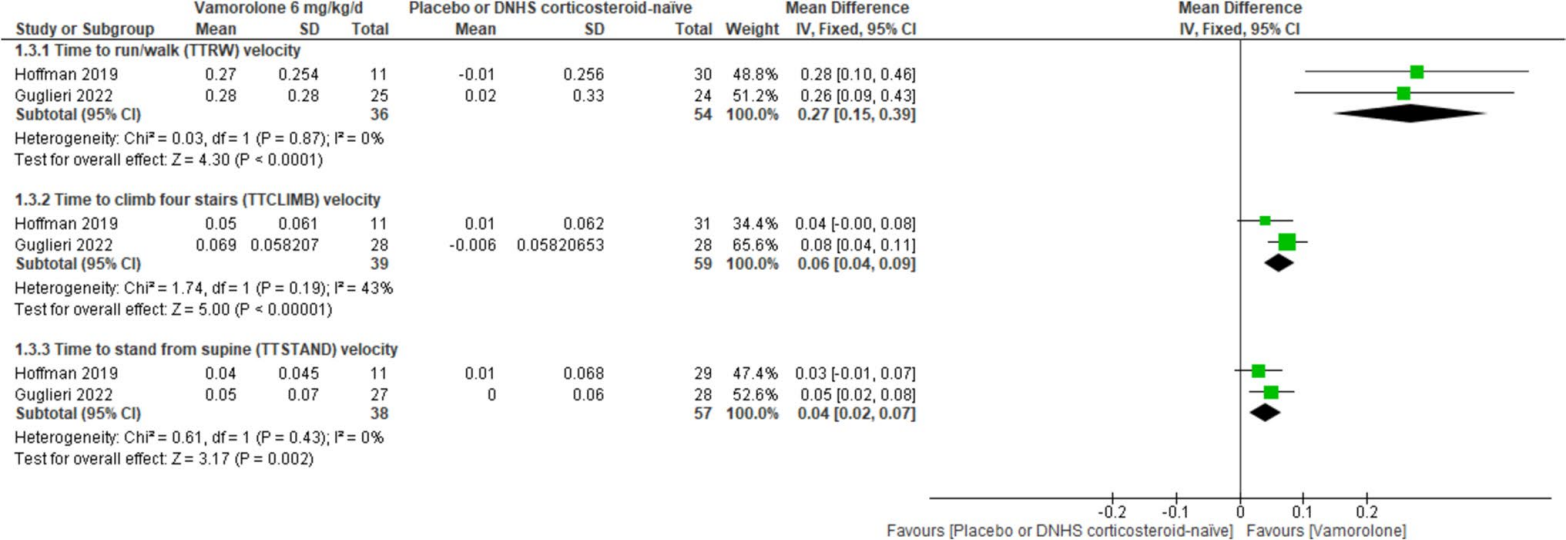
Forest plot comparing the efficacy outcomes of vamorolone 6 mg/kg/d and placebo or DNHS corticosteroid-naïve at 24 weeks

**Fig. 3:**
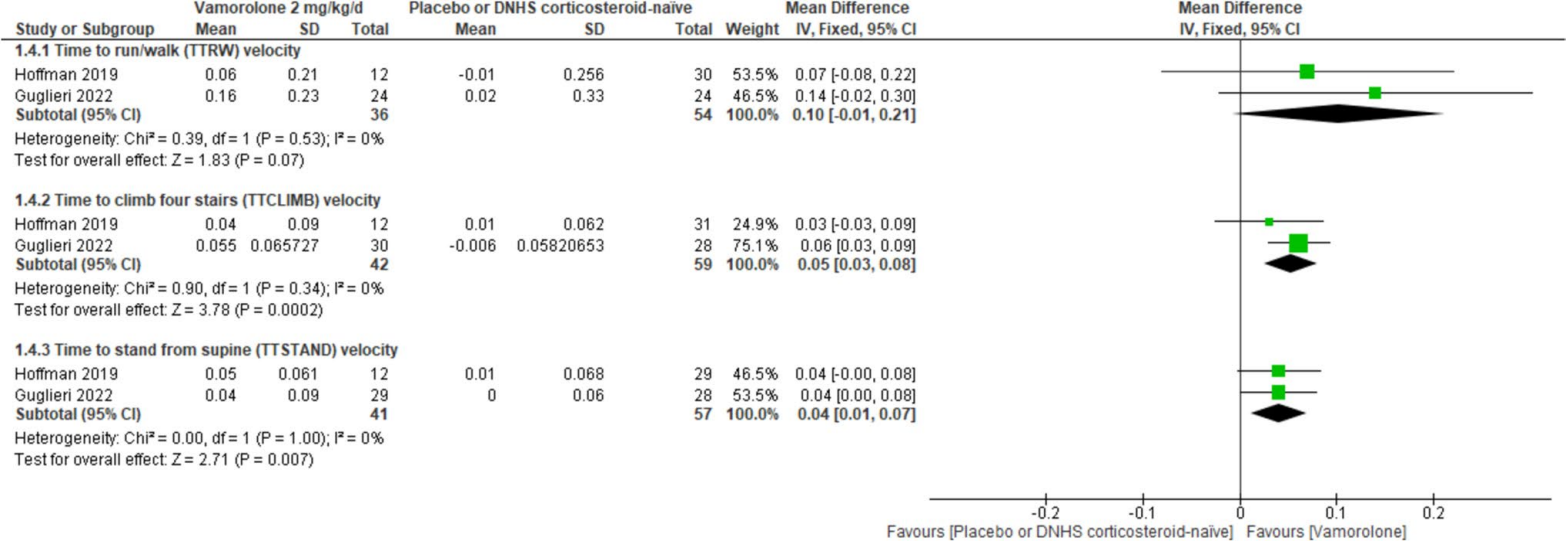
Forest plot comparing the efficacy outcomes of vamorolone 2 mg/kg/d and placebo or DNHS corticosteroid-naïve at 24 weeks

Reports of data were sufficient to conduct a meta-analysis in two studies providing data from a total of 210 patients for TTRW velocity, TTCLIMB velocity, and TTSTAND velocity at 24 weeks. The overall mean difference favored the vamorolone groups over placebo or corticosteroid-naïve groups for 2.0, and 6.0 mg/kg/day doses in TTCLIMB velocity, and TTSTAND velocity and for 6.0 mg/kg/day dose only in TTRW velocity (Figs. 2 and 3).

The vamorolone 2.0 mg/kg/day and 6.0 mg/kg/day groups showed a statistically significant dose-related improvement from baseline to 6 months and 18 months for 6MWT, TTRW velocity, TTCLIMB velocity, TTSTAND velocity and NSAA score, respectively. However, at 30 months, the improvement from baseline disappeared except for TTCLIMB velocity (Table 3).

**Table 3:** Summary of the outcomes in each included study

Outcome		Dose	Conklin 2018 [9]	Hoffman 2019 [21]	Smith 2020 [8]	Mah 2022 [20]	Guglieri 2022 [22]
6MWT	Improvement from baseline	2.0 and 6.0 mg/kg/d	NR	Significant	Significant	No statistically significant improvement	Significant
	Comparison with placebo or steroid-naïve			NR	NR	NR	Statistically significant difference favoring vamorolone
	Comparison with steroid			NR	NR	NR	NR
TTRW velocity	Improvement from baseline	2.0 and 6.0 mg/kg/d	NR	Significant	Significant	No statistically significant improvement	Significant
	Comparison with placebo or steroid-naïve			Statistically significant difference favoring vamorolone	Statistically significant difference favoring vamorolone	NR	Statistically significant difference favoring vamorolone
	Comparison with steroid			NR	NR	No statistically significant difference	NR
TTCLIMB velocity	Improvement from baseline	2.0 and 6.0 mg/kg/d	NR	Significant	Significant	statistically significant improvement	Significant
	Comparison with placebo or steroid-naïve			No statistically significant difference	No statistically significant difference	NR	Statistically significant difference favoring vamorolone
	Comparison with steroid			NR	NR	No statistically significant difference	NR
TTSTAND velocity	Improvement from baseline	2.0 and 6.0 mg/kg/d	NR	Significant	Significant	No statistically significant improvement	Significant
	Comparison with placebo or steroid-naïve			Statistically significant difference favoring vamorolone	No statistically significant difference	NR	Statistically significant difference favoring vamorolone
	Comparison with steroid			NR	NR	No statistically significant difference	NR
NSAA score	Improvement from baseline	2.0 and 6.0 mg/kg/d	NR	Significant	Significant	No statistically significant improvement	Significant
	Comparison with placebo or steroid-naïve			NR	NR	NR	Statistically significant difference favoring vamorolone
	Comparison with steroid			NR	NR	No statistically significant difference	NR
Mean height percentile for age	Change from baseline	2.0 and 6.0 mg/kg/d	NR	NR	Significantly increased	Significantly increased	Significantly increased
	Comparison with placebo or steroid-naïve				Statistically significant difference (higher with vamorolone)	NR	NR
	Comparison with steroid				Statistically significant difference (higher with vamorolone)	Statistically significant difference (higher with vamorolone)	Statistically significant difference (higher with vamorolone)
Mean BMI z-score	Change from baseline	2.0 and 6.0 mg/kg/d	NR	Significantly decreased	Significantly decreased	Significantly decreased	Significantly increased
	Comparison with placebo or steroid-naïve			NR	Statistically significant difference (higher with vamorolone)	NR	NR
	Comparison with steroid			Statistically significant difference (lower with vamorolone)	No statistically significant difference	No statistically significant difference	No statistically significant difference
Osteocalcin	Change from baseline	2.0 and 6.0 mg/kg/d	Significantly decreased	Significantly increased	NR	NR	Significantly increased
	Comparison with placebo or steroid-naïve		NR	NR			NR
	Comparison with steroid		NR	NR			Statistically significant difference (high with vamorolone)
P1NP	Change from baseline	2.0 and 6.0 mg/kg/d	Significantly decreased	No significant change	NR	NR	Significantly increased
	Comparison with placebo or steroid-naïve		NR	NR			NR
	Comparison with steroid		NR	NR			Statistically significant difference (high with vamorolone)
CTX1	Change from baseline	2.0 and 6.0 mg/kg/d	Signific antly decreas ed	Significantly increased	NR	NR	Significantly increased
	Comparison with placebo or steroid-naïve		NR	NR			NR
	Comparison with steroid		NR	NR			Statistically significant difference (higher with vamorolone)
Morning cortisol	Change from baseline	2.0 and 6.0 mg/kg/d	Signific antly decreas ed	Significantly decreased	NR	NR	Significantly decreased
	Comparison with placebo or steroid-naïve		NR	NR			NR
	Comparison with steroid		NR	NR			Statistically significant difference (higher with vamorolone 2 mg, but not 6 mg)
ACTH levels	Change from baseline	2.0 and 6.0 mg/kg/d	Significantly decreased	Significantly decreased	NR	NR	Significantly decreased
	Comparison with placebo or steroid-naïve		NR	NR			NR
	Comparison with steroid		NR	NR			Statistically significant difference (higher with vamorolone 2 mg, but not 6 mg)
Fasting serum insulin	Change from baseline	2.0 and 6.0 mg/kg/d	Significantly increased	Significantly increased	NR	NR	NR
	Comparison with placebo or steroid-naïve		NR	NR			
	Comparison with steroid		NR	NR			
Fasting serum glucose	Change from baseline	2.0 and 6.0 mg/kg/d	No Signific ant change	Significantly decreased	NR	NR	NR
	Comparison with placebo or steroid-naïve		NR	NR			
	Comparison with steroid		NR	NR			
Serum creatinine kinase	Change from baseline	2.0 and 6.0 mg/kg/d	Significantly decreased	NR	NR	NR	NR
	Comparison with placebo or steroid-naïve		NR				
	Comparison with steroid		NR				

*6MWT,* Six minutes walking test velocity (meters/second); *TTRW,* Time to run/walk 10 m velocity (meters/second); *TTCLIMB,* Time to climb four stairs velocity (event/second); *TTSTAND,* Time stand from supine velocity (event/second); *NSAA,* North Star Ambulatory Assessment score (of 34); *BMI z-score,* Body mass index z score; *P1NP,* procollagen type I propeptides (ng/mL); *CTX1,* type 1 collagen cross-linked C-telopeptide (pg/mL)); *ACTH,* Adrenocorticotropic hormone levels (μg/dL)); *NR,* Not reported

### Safety

*Growth measures (mean height percentile for age and mean BMI z-score):* At 24 weeks, the prednisone group showed a decline in height and a delay of linear growth, whereas the vamorolone group did not. However, both groups had similar overall BMI z-score gains. At 18 months, the vamorolone group showed an increase of the mean BMI z-score, but no significant differences in height percentile or BMI z-score compared to the corticosteroid-naïve participants. At 30 months, there was no evidence of growth delay in the vamorolone group, but there was a significant difference in the mean height percentile change between participants receiving high-dose vamorolone and those treated with glucocorticosteroids in the DNHS (Table 3).

*Biomarkers of bone turnover (Osteocalcin, P1NP, and CTX1):* Vamorolone decreased CTX1 and P1NP at different doses, but the dose-effect relationship of P1NP was unclear. Osteocalcin was increased at lower doses but decreased at the highest dose of vamorolone. Prednisone treatment resulted in marked reductions of these markers, whereas vamorolone did not. There were no assessments of these biomarkers at 18 or 30 months (Table 3).

*Adrenal axis suppression (Morning cortisol and ACTH levels):* Treatment with vamorolone showed a dose-related suppression of serum morning cortisol and ACTH when measured at two weeks and 24 weeks. These findings were consistent with both short- and long-term adrenal suppression as illustrated in Table 3.

*Biomarkers of insulin resistance (fasting serum insulin and serum glucose):* The mean changes from baseline to 2 weeks of fasting glucose and insulin were not significant for any dosage. However, at 24 weeks, a significant change from baseline was observed for fasting glucose in the 2 and 6 mg/kg groups and for fasting insulin in the 6 mg/kg group.

*Serum creatine kinase (biomarker of muscle activity):* Serum creatine kinase was only measured at four weeks. The mean change from baseline to four weeks showed a significant decline of serum CK in the 2 and 6 mg/kg groups.

*Treatment emergent adverse event (TEAE):* At four weeks, 46 TEAEs were observed among the 48 participants. The highest incidence was for upper respiratory tract infections followed by diarrhea. Of these, only eight were considered as possibly related to the study drug. No serious adverse events were observed. At 24 weeks, 42 TEAEs were observed among the 48 participants. The most common adverse events were upper respiratory tract infections followed by pyrexia. One patient in the 0.75 mg/kg/d group and 2 patients in the 6.0 mg/kg/d group had a total of 4 treatment-related serious adverse events. However, none of the serious adverse events was considered to be related to the drug investigated. When compared to the placebo and prednisone groups, the proportion of individuals who reported at least one TEAE was comparable between groups. The total number of TEAEs was lowest in the placebo group, largest in the prednisone group, and intermediate in the 2 vamorolone groups. Of the total number of reported TEAEs from the start of VBP15-002 until 18 months, 402 were unrelated to vamorolone, 37 were remotely connected, 29 were potentially associated, 11 were probably related, and 3 were definitely related. 10/14 adverse effects (AEs) associated with vamorolone were weight gain, 2/14 were an increase of appetite, one was the development of Cushingoid characteristics, and one was irritability. At 30 months, all 46 participants experienced at least one TEAE. A TEAE of weight gain led to the reduction of the vamorolone dose from 6 mg/kg/d to 2 mg/kg/d in 10 individuals. In six of these patients, the weight returned to normal after dose reduction. Six participants suffered a total of 7 clinical fracture events. However, according to the investigators, these TEAE were unrelated to the study medication.

## Discussion

While our systematic review and meta-analysis revealed an overall positive efficacy profile of the treatment it also highlighted relevant safety concerns, ultimately demonstrating the need for further well-designed research on this promising treatment. Vamorolone showed a statistically significant improvement in 6MWT, TTRW velocity, TTCLIMB velocity, TTSTAND velocity, and NSAA score compared to placebo when taken at 2 and 6 mg/kg/day for 24 weeks (except for TTRW velocity at 2 mg/kg/d). At 18 months, both doses still showed improvement; however, at 30 months follow-up the improvement no longer favored the vamorolone groups except for TTCLIMB velocity. In terms of safety, the results of this systematic review suggest that while vamorolone therapy does not impair growth, there is a risk of weight gain. Vamorolone showed dose-dependent adrenal suppression, which was consistent both short and long-term. Furthermore, there was a significant decrease of the markers of bone resorption and formation at two weeks, but a significant increase until week 24. There was a decline of serum creatine kinase in the 2 and 6 mg/kg groups. In addition, we observed an increase of the markers of insulin resistance at 24 weeks. Vamorolone had some adverse effects, but most were not considered serious and were not related to treatment. In participants who experienced weight gain, this adverse effect was ameliorated by reducing the dose of vamorolone. One of the earliest molecular changes observed in DMD patients is the activation of cell damage pathways related to NFκB [23]. Prednisone and other glucocorticoids diffuse across the cell membrane, bind to the cytoplasmic nuclear hormone receptor (glucocorticoid receptor (GR)), and then translocate to the nucleus as a receptor-ligand complex. This complex binds to a specific DNA sequence, the glucocorticoid response element (GRE), and thus transactivates target genes associated with broad-spectrum anti-inflammation. It further suppresses the transcription of NF-B, the master regulator of inflammation (“transrepression”) [24, 25]. These processes underlie the beneficial effects of glucocorticosteroids in DMD.

In contrast, many adverse effects (e.g. muscle atrophy, growth retardation) of traditional steroids are caused by the GR ligand complex binding to other target genes and repressing their transcription ("cis-repression") [26]. Vamorolone retains the transrepression and thus the anti-inflammatory properties of traditional steroids, but at the same time—due to its different molecular structure—avoids the transactivation and cis-repression of the target genes that cause the negative side effects of other steroids [27, 28].

In addition, many corticosteroids, including prednisone and deflazacort, are agonists at the mineralocorticoid receptor, leading to increases in blood volume and blood pressure. Conversely, vamorolone is a potent antagonist at the mineralocorticoid receptor, similar in its activity to eplerenone and spironolactone [28].

In summary, vamorolone does not activate the transcription of genes associated with GRE binding and activation, while retaining anti-inflammatory properties through NFκB inhibition. Unlike traditional steroids, it is a potent inhibitor at the mineralocorticoid receptor and has excellent membrane stabilizing properties [26, 29].

The findings of this systematic review suggest that vamorolone may be a valid treatment option for patients with DMD. Vamorolone seem to improve several efficacy outcomes, such as 6MWT, TTRW velocity, TTCLIMB velocity, TTSTAND velocity, and NSAA score, compared to the placebo when taken at 2.0 and 6.0 mg/kg/day for 24 weeks. At 18 months, both doses still showed statistically significant improvement, although at 30 months the improvement was no longer in favor of the vamorolone groups, except for TTCLIMB velocity.

The most important issues regarding the treatment of DMD patients are long-term efficacy and safety [30, 31]. Over time, the efficacy of conventional steroids may decrease in some patients [32]. This may be due to four main factors: 1.) Resistance to steroids: As the disease progresses, steroids may become less effective over time. 2.) Tolerance: Long-term use of steroids may require higher doses to achieve the same effect. 3.) Side effects: Adverse effects include weight gain, mood changes, osteoporosis, metabolic disorders, increased risk of infection. 4.) Disease progression: The natural history of DMD causes a progressive loss of muscle mass and strength despite treatment with steroids [33, 34].

A potential advantage of vamorolone over corticosteroids is that it may not share the same efficacy limitations with long-term use. It has been shown that the anti-inflammatory effects of vamorolone are maintained over a 2-year period, without the loss of efficacy that has been observed with conventional steroids [8, 20]. This is thought to be due to the fact that vamorolone is designed to target specific inflammatory pathways in the body, which may help to avoid the development of resistance or tolerance associated with conventional steroids [6, 27, 28]. However, at 30 months, the observed improvements no longer favored the vamorolone groups, except for TTCLIMB velocity [20]. Therefore, it remains unclear whether vamorolone provides long-term sustained efficacy beyond 30 months.

Osteocalcin and P1NP are markers of bone formation and correlate with bone density and geometry [35]. They remain unchanged with vamorolone, but decrease with glucocorticoid therapy [36]. CTX1is a marker of bone resorption and increases with conventional glucocorticoid treatment [37, 38]. Osteoporosis is a common side effect of glucocorticoid medication, and 30% to 50% of DMD patients on long-term steroid therapy develop fractures [39]. The highest risk of osteoporosis has been shown to occur with prednisone doses of 15 mg/day or higher [40]. The normal values of the bone turnover markers in DMD patients on vamorolone imply an improved safety profile of vamorolone for bone health [41, 42]. Clinically, this is supported by the absence of retardation in patients receiving vamorolone.

It has been shown in mice that a 11-hydroxysteroid dehydrogenase is required for the development of steroid-induced bone disease [43, 44]. However, vamorolone is not a substrate for these enzymes, because it lacks the 11-hydroxysteroid moiety [22]. This may explain why the vamorolone-treated groups had a better bone biomarker profile than the corticosteroid-treated groups. However, the risk of weight gain associated with glucocorticosteroids may also apply to vamorolone-treated patients [41].

The use of traditional glucocorticosteroids causes a strong, widespread, acute and long-term inhibition of the hypothalamic-pituitary-adrenal axis, which also occurred with vamorolone [45]. An emerging adrenal insufficiency can be treated with supplementary glucocorticosteroids [46]. Furthermore, the X chromosomal location of the DMD and congenital adrenal hypoplasia genes are adjacent, suggests a possible mechanistic connection between DMD and adrenal insufficiency [22, 47, 48]. Vamorolone may be effective in treating adrenal insufficiency without the safety concerns associated with glucocorticosteroids requires investigation. Prospective well-designed research is required in this context.

The pathologic biomarkers of insulin resistance suggest that vamorolone shares this adverse event with the traditional steroids used in DMD. This study also showed that vamorolone decreased serum CK in a manner similar to conventional steroids [49]. In practice, these findings may have important implications for patients with DMD. Vamorolone has a different mechanism of action than conventional corticosteroid anti-inflammatory drugs, which may result in fewer side effects and better safety outcomes. Furthermore, the potential of vamorolone to treat adrenal insufficiency without the safety concerns associated with glucocorticosteroids in patients with DMD requires further study. Research is needed to further assess the long-term safety and efficacy of vamorolone and to determine its role in the treatment of DMD.

For clinicians and elderly patients, not only evidence of long-term efficacy, but also robust data on whether vamorolone prevents fractures and scoliosis, and whether it is effective on disease progression in non-ambulatory patients with DMD, are important issues for further study and represent an important knowledge gap.

It is noteworthy that a recent meta-analysis found similar results supporting the efficacy of vamorolone in the treatment of DMD. Our approach to data synthesis differed from this work because we excluded open-label extension trials in identical or overlapping study populations from the meta-analysis [50]. While this approach may not be superior to the aforementioned review, it may provide an accurate reflection of the current, albeit limited, body of evidence.

There are limitations of this systematic review and metaanalysis which include the small sample size of the individual articles and the limited number of studies which could be included in the meta-analysis. The duration of action of vamorolone and access to the medication are important issues in clinical practice. Moreover, due to the short follow-up period of the included trials, it was not possible to draw definitive conclusions regarding the long-term efficacy of vamorolone.

Confirmatory well-designed randomized controlled trials are needed to asses and reaffirm a beneficial efficacy and safety profile of vamorolone for DMD [51].

## Conclusion

We provide meta-analytic evidence suggestive of a potentially favorable safety and efficacy profile of vamorolone as a treatment option for patients with DMD. Further well-designed, controlled interventional studies and real-world data analysis are needed to further explore the net clinical value of this treatment.

## Supplementary Information

Below is the link to the electronic supplementary material.

Supplementary file1 (PDF 235 KB)

## Data Availability

The datasets used and/or analyzed during the current study are available as MS Excel files (.xlsx) and RevMan file (.rm5) from the corresponding author upon reasonable request.
